# Different delivery mechanisms for insecticide-treated nets in rural Burkina Faso: a provider's perspective

**DOI:** 10.1186/1475-2875-9-352

**Published:** 2010-12-04

**Authors:** Claudia Beiersmann, Manuela De Allegri, Justin Tiendrebéogo, Maurice Yé, Albrecht Jahn, Olaf Mueller

**Affiliations:** 1 Institute of Public Health, Ruprecht-Karls-University Heidelberg, INF 324, 69124 Heidelberg, Germany; 2 Centre de Recherche en Santé de Nouna, BP 34, Nouna, Burkina Faso

## Abstract

**Background:**

Insecticide-treated nets (ITNs) have been confirmed to be a very effective tool in malaria control. Two different delivery strategies for roll-out of ITN programmes have been the focus of debate in the last years: free distribution and distribution through commercial marketing systems. They are now seen as complementary rather than opponent. Acceptance of these programmes by the community and involved providers is an important aspect influencing their sustainability. This paper looks at how providers perceived, understood and accepted two interventions involving two different delivery strategies (subsidized sales supported by social marketing and free distribution to pregnant women attending antenatal care services).

**Methods:**

The interventions took place in one province of north-western Burkina Faso in 2006 in the frame of a large randomized controlled ITN intervention study. For this descriptive qualitative study data were collected through focus group discussions and individual interviews. A total of four focus group discussions and eleven individual interviews have been conducted with the providers of the study interventions.

**Results:**

The free distribution intervention was well accepted and perceived as running well. The health care staff had a positive and beneficial view of the intervention and did not feel overwhelmed by the additional workload. The social marketing intervention was also seen as positive by the rural shopkeepers. However, working in market economy, shopkeepers feared the risk of unsold ITNs, due to the low demand and capacity to pay for the product in the community.

**Conclusion:**

The combination of ITN free distribution and social marketing was in general well accepted by the different providers. However, low purchasing power of clients and the resulting financial insecurities of shopkeepers remain a challenge to ITN social marketing in rural SSA.

## Background

Malaria is still one of the leading causes of morbidity and mortality in Africa [1,2]. In the last years insecticide-treated nets (ITNs) have been confirmed to be a very effective tool in malaria control [3]. Estimations of the percentage of African households owning at least one insecticide-treated net (ITN) are increasing. The percentage of children using an ITN, however, is still below the World Health Assembly target of 80% [4]. Therefore, scaling-up ITN coverage in SSA is a top priority for both national and international health agencies.

The last few years have been characterized by a sharp debate on which delivery mechanisms are best suited to facilitate rapid and substantial increases in ITN coverage. This debate has focused on two different delivery strategies: free distribution and distribution through commercial marketing systems. The first strategy arguments that due to prevailing poverty ITNs should be considered as a public good like vaccines and consequently be provided through the public sector free of charge [5-10]. Others criticize this approach as being not sustainable due to its intrinsic need for excessive donor investment and rather favour the strengthening of commercial markets [9,11-14]. Recently the two positions approached each other. Most authors and policy makers now believe in a mixture of both, seeing them as complementary rather than opponent [15-19], calling for complementing market sales with highly subsidized and/or free distribution programmes [14-16,20]. Of high interest in the ITN scaling up process is, besides effectiveness and feasibility, also the cost-effectiveness and financing mechanisms. Cost-effectiveness of this intervention has been shown by a number of studies [7,21-25]. However, conclusions to decide which delivery mechanism is most cost-effective aren't easy to make, as they vary widely in scale, target populations and methods [26].

Acceptance of these programmes by the community and involved providers is an important aspect influencing their sustainability. There are only a handful of studies exploring how different delivery strategies have been perceived by communities [27,28]. Excluding a process-focused paper [29] studies looking at the provider's perspective are nonexistent. This paper aims to fill this gap in knowledge by investigating how providers perceived, understood and accepted two interventions involving two different delivery strategies (subsidized sales supported by social marketing and free distribution to pregnant women attending antenatal care services (ANC). In this study, the term provider is used to refer not only to health care providers, but also to all the players involved in the organisation and management of the two interventions. The interventions took place in one province of north-western Burkina Faso in 2006. This paper focuses on results based on individual interviews and focus group discussions.

## Methods

### Study area

The study took place in the Nouna Health District (NHD), which is equivalent to the Kossi province, located in northwestern Burkina Faso, approximately 300 km from the capital Ouagadougou. The NHD covers a population of around 311,000 people living in some 300 villages and Nouna town, the capital of the Kossi province [30]. At the time of the beginning of the study, the NHD counted 24 village-based first-line health care facilities (CSPS, Centre de Santé et Promotion Social), one urban CSPS in Nouna town, and the Nouna district hospital [31]. The CSPS were responsible for ANC services, with the exception of PMTCT services which at the time were only available at the Nouna district hospital. People living in this savannah area are from different ethnic groups and work mainly as subsistence farmers. Malaria is endemic, but also highly seasonal. Most malaria transmission takes place during and shortly after the rainy season which usually lasts from June until October [32].

### ITN interventions in the study area

This study was embedded within a community-based cluster-randomized controlled trial (trial registration: Current Controlled Trials Ltd. ISRCTN07985309). Details of the trial are described elsewhere [33]. In brief, the NHD was divided into 24 clusters according to the health facilities and their catchment areas. These 24 clusters were randomized to receive either intervention A (social marketing of ITNs to the general population plus free distribution of ITN to pregnant women attending ANC services, 12 clusters) or intervention B (only social marketing of ITN to the general population, 12 clusters). One additional cluster, the urban CSPS of Nouna town and its catchment area, was pre-assigned to intervention B. The primary outcome of the trial was ITN coverage at the household level - measured through annual household surveys.

The interventions were carried out by the national malaria programme (PNLP) together with its partners - the local Health District and Population Services International (PSI), a non-governmental organisation (NGO) active in the field of malaria control in Burkina Faso. The PNLP was responsible for the initial procurement of ITNs, which could be purchased thanks to a Global Fund initiative, and for their subsequent allocation to the NHD team and to PSI. The ITN product was a deltamethrin-impregnated white family-size long lasting ITN (PermaNet, Vestergaard Frandsen, Denmark) with the brand name Serena.

The NHD team was in charge of the free distribution of the ITNs to pregnant women who attended ANC at the local CSPS. The NHD received an initial stock of 5,000 ITNs. This amount had been estimated to cover need given the expected pregnancy incidence. Demand, however, exceeded expectations, so the original stock was later complemented with an additional 1,000 ITNs. At the beginning of the intervention, the twelve selected CSPS were provided with an initial stock of ITNs, depending on the size of their catchment area and the expected number of pregnancies in the relevant communities. Health staff could refill their stock at central deposit of the District located in Nouna town at any point in time. They were expected to transport the ITNs to their facilities on their motorbikes.

Already prior to the launch of the intervention, the staff of the CSPS received training on the product, its function, the distribution modalities, and the details of the trial design. The training was provided by a team of PNLP and District staff and by two researchers from the Nouna Health Research Centre (CRSN), a governmental research institute situated in Nouna town and the institution in charge of managing the trial.

Nurses and midwives were instructed to hand out one ITN per pregnant women, independently from the stage of pregnancy, at her first contact with ANC services following the beginning of the intervention. They were further instructed to counsel women to sleep under the ITN throughout the pregnancy and to place their newborn babies underneath afterwards.

To limit the risk that women could receive additional ITNs at later visits, ITN delivery was recorded both on the ANC register and on the ANC booklet of the single women. To minimize leakages and to ensure that ITNs were delivered correctly, a close supervision system was set in place. A supervisor from the central District team visited all concerned CSPS every three months to check the coherence between the amount of ITNs received and the amount dispensed. On such occasions, the supervisor also assessed whether the intervention was running smoothly and whether the message on the protection offered by ITNs against malaria was delivered correctly.

PSI implemented the social marketing intervention, which took place according to the modalities normally employed by PSI in the country. 15,000 Serena ITNs were sold to a wholesaler in Nouna town who had been identified by PSI for a price of 950 FCFA each (Franc Communauté Financière d'Afrique; 1 Euro ~650 FCFA). Shopkeepers could buy Serena from the wholesaler for a price of 1,075 FCFA each. The final customer was expected to pay 1,500 FCFA per ITN. The revenues from sales were re-invested by PSI to support its social marketing campaign.

In August 2006, PSI conducted a social marketing campaign to support their sales and create demand for Serena. This campaign consisted of village-based information, education and communication (IEC) activities, specific local radio spots (in addition to already existing national radio and TV messages), and poster exhibition, which informed about malaria and the possibility of prevention via ITNs. Additionally PSI conducted ITN promotion visits to potential sellers in rural villages. A large number of villages were visited to convince shopkeepers to participate in the intervention, through buying and then reselling Serena ITNs. There was the possibility to purchase a teaser of three to five Serena ITNs from the staff members of PSI conducting the ITN promotion visits. For further Serena ITN procurement, the shopkeepers were referred to the wholesaler in Nouna town to fill their stocks.

### Study design and procedures

Fieldwork was conducted in cooperation with the CRSN. The study relied on focus group discussions (FGD) and individual interviews (II) as data collection methods to gain insight into how providers perceived, understood and accepted the two delivery interventions. A total of four FGDs and eleven IIs were conducted over a period of seven weeks between May and July 2007.

The four FGDs were conducted with all the Head Nurses (ICP, infirmiers chef de poste) and antenatal care providers (either a lower trained nurse or a midwife) of the CSPS of both intervention areas A and B (if not stated otherwise the expression ‚CSPS staff' refers to those two staff categories). The staff of intervention area A was responsible for the free ITN distribution through ANC services.

Individual interviews where conducted with:

• the district-level pharmacist, responsible for the overall planning and coordination of the free distribution and in charge of the overall ITN stock for the free distribution (n = 1)

• the director of PSI, responsible for the overall planning and coordination of the social marketing intervention (n = 1)

• two promoters of the social marketing of PSI, responsible for the promotion of the social marketing intervention to the wholesaler, shopkeepers and the communities (n = 1)

• the wholesaler in Nouna town, responsible for the promotion and selling of the Serena ITNs to rural shopkeepers and the community population (n = 1)

• rural shopkeepers who agreed to sell Serena ITNs in their shops (n = 3)

• rural shopkeepers who had not agreed to sell Serena ITNs in their shops (n = 3)

• the director of the National Malaria Programme (*Programme National de Lutte contre le Paludisme, PNLP*), responsible for the initial procurement of ITNs and for their subsequent allocation to the NHD and to PSI (n = 1)

The participating providers were purposely sampled as they were the ones involved in the processes of planning, implementation and realization of these interventions. For convenient purposes the interviews with the rural shopkeepers were conducted in villages where FGDs with the community had taken place in the framework of the overall community-based cluster-randomized controlled trial. For details on how these villages were selected see Beiersmann *et al *[27]. Table 1 provides an overview of the respondents and the relevant data collection methods.

**Table 1:** Overview of respondents and relevant data collection method

Data collection method (venue/facilitator/language)	Respondents
Focus group discussion (n = 1) Nouna town/interviewers^1^/French	ICP^2^s of the CSPS^3 ^intervention area A

Focus group discussion (n = 1) Nouna town/interviewers/French	ICPs of the CSPS intervention area B

Focus group discussion (n = 1) Nouna town/interviewers/French	Antenatal care providers^4 ^of the CSPS intervention area A

Focus group discussion (n = 1) Nouna town/interviewers/French	Antenatal care providers of the CSPS intervention area B

Individual interview (n = 1) Nouna town/first author/French	District level pharmacist

Individual interview (n = 1) Ouagadougou/first author/French	Director of PSI

Individual interview (n = 1) Ouagadougou/first author/French	Two promoters of the PSI social marketing

Individual interview (n = 1) Nouna//interviewer/Dioula^5^	Wholesaler in Nouna town

Individual interview (n = 3) Village/interviewer/Dioula	Rural shopkeepers who agreed to sell Serena ITN

Individual interview (n = 3) Village/interviewer/Dioula	Rural shopkeepers who did not agree to sell Serena ITN

Individual interview (n = 1) Ouagadougou/first author/French	Director of PNLP^6^

^1^Interviewer refers here to trained interviewers of the CRSN

^2^ICP stands for Infirmier Chef de Poste, i.e. the head nurse in charge of the village-based first-line health care facility

^3^CSPS stands for Centre de Santé et Promotion Social, village-based first-line health care facilities

^4^The antenatal care provider differs from CSPS to CSPS. In some CSPS, antenatal care is provided by a midwife; in others, by the ICP himself; in others yet, by a lower level nurse

^5^Dioula is the local lingua franca

^6^PNLP stands for Programme National de Lutte contre le Paludisme, i.e. the National Malaria Programme

The FGD were formed on the basis of whether the participants had been working in intervention area A or intervention area B (see Table 1). The number of participants in the FGD varied between 12 and 14. Discussion focused on nurses and midwives' perceptions of the free distribution of ITNs through ANC services and their involvement in the intervention, including their judgment of the impact of the programme on their overall service provision. The individual interviews with PSI, the wholesaler and the shopkeepers focused on their overall perception of the social marketing campaign, the implementation process, and the role that they had played in it. The individual interview with the director of the PNLP focused on its experience with the overall management of the two interventions.

All interview guides were initially drafted by the first author and discussed with the second and third author at various stages of their development. The first and third author trained the interviewers so that every body was familiar with the specificities of the study and the interview guide. The individual interviews with the rural shopkeepers and the wholesaler in Nouna town were conducted by the interviewers in Dioula, the local lingua franca. All other individual interviews and the FGDs were conducted in French. The interviews and FGDs were tape recorded with permission of the participants, later verbatim transcribed and where necessary translated into French by the interviewers themselves.

### Data analysis

The first author analysed the data through content analysis using software AtlasTi 5.2 [34]. She coded the data material using an initial set of codes developed on the basis of the interview guide. Whilst progressing through the material, she complemented this set of codes with additional codes as information emerged from the data. For triangulation purposes, the second author analysed a selection of transcripts to compare and converge findings.

### Ethical aspects

Approval for the ITN trial within which this study was embedded was obtained by the Ethics Committee of the Heidelberg University Medical School and the local Ethics Committee in the town of Nouna (that is the institutional review board of the CRSN).

Before each FGD or interview, the aim of the study was described and oral informed consent was sought from the participant(s). Confidentiality was assured at all stages of the study. Permission was asked for tape-recording.

## Results

For purposes of clarity, the results section will first report on findings relevant to the free distribution (FD) intervention and then on findings relevant to the social marketing (SM) intervention. Findings are illustrated using verbatim quotations.

### Free distribution

The CSPS staff of intervention area A as well as intervention area B was aware of the FD intervention, its aims, and objectives. The CSPS staff of intervention area A where the FD was implemented was also well aware of the relevant working processes concerning this intervention.

### Opinions and attitudes towards the FD intervention and its influence on the success of the programme

The CSPS staff appreciated the free distribution (FD), staff in intervention area A as well as staff in intervention area B. Respondents considered the FD as a form of aid for the poor population and especially for pregnant women. The CSPS staff of intervention area A reported that the FD intervention went well. They perceived the ITN product Serena as of good quality and in their view the FD was well accepted by the population. They had the impression that it increased the utilization of antenatal care (ANC) services. The ICPs in intervention area A stated to have observed that malaria incidence among pregnant women decreased and that, due to the incentive of receiving an ITN, women visited ANC earlier in their pregnancy.

R: "The distribution...is running well. The population is very motivated. People appreciate very much this distribution. And it improves also ANC attendance in those CSPS that distribute the ITNs for free... but there is also a considerable reduction in pregnant women falling sick with malaria." (FGD ICPs intervention area A)

R: "...since we have had the ITNs there are others [other pregnant women] who come in the second month of their pregnancy even. Before, we saw them in their seventh or sixth month, but now from about their 3^rd ^month on, they come." (FGD ICPs intervention area A)

Respondents observed that there are pregnant women who received a free Serena ITN at their first ANC visit but who do not come back then for their second and/or third ANC visit. They suggested solving this problem by giving out the ITN not at the first, but at a later ANC visit.

R: "If the [pregnant] woman takes it [the ITN]) at her first ANC visit, she does not come back. We have noticed that the first ANC visit surmounts the other ones." (FGD ICPs intervention area A)

Respondents indicated that the FD intervention increased their workload. However, they did not complain of such an increase. On the contrary, the CSPS staff in intervention area A desired a project continuation, while the CSPS staff in intervention area B wished for an expansion of the project.

R: "We cannot even imagine stopping the free distribution. We should always continue to distribute the ITNs... I think we should continue to distribute [them] in all of the CSPS, in the entire district." (FGD antenatal care providers intervention area A)

R: "The project is creditable. I think that it is a good thing, but I wish to see it being expanded." (FGD antenatal care providers intervention area B)

One problem emerged due to the randomized study design. Respondents of intervention area B (no FD) reported that pregnant women did ask for free ITNs in their CSPS or switched to a CSPS with free distribution. Correspondingly respondents of intervention area A (with FD) stated that there were women coming from other areas to get a free ITN. Respondents further admitted that they found it difficult to make women understand that they were not eligible for a net.

R: "... there are women from other CSPS who come for ANC to the CSPS with free distribution. It is frustrating to give an ITN to some and not to others ... actually we have had difficulties to make woman understand this. If we could expand the programme...." (FGD ICPs intervention area A)

### Potential abuse of the ITNs

The CSPS staffs view the distribution process of the Serena ITNs as transparent.

R: "A woman can not come saying that she did not receive [an ITN] although she already received one. If she has received one, the moment we look into her carnet we see it, we know it also from the facility registry. This helps us to control the release of the ITNs. (FGD antenatal care providers intervention area A)

### Factors hindering the smooth realization of the programme and areas for improvement

Respondents of intervention area A asked for more feedback on how the project was running, but also specifically on their work and performance.

R: "... we realize ... that there is a supervisor who visits the CSPS. ... we expected some feedback, since this intervention concerns the CSPS. We do not know what has worked out and what has not worked out...." (FGD ICPs intervention area A)

Respondents in intervention area A identified stock ruptures as the most important problem they faced in relation to the intervention. They had to make sure that there are always enough ITNs available for the free distribution through their ANC services. They complained that, when filling their stock at the district pharmacist in Nouna town, they were given an amount of ITNs that was often not sufficient to cover the number of pregnant women attending the ANC services up to their next travel to Nouna. They stated that there was no budget for additional travels to Nouna just for the purpose of refilling their stock. They furthermore mentioned that supplying themselves with larger amounts of ITNs was also difficult because of limited transport capacity and bad road conditions.

R: "... For instance, for the CSPS of Doumbala,, according to the ITN distribution plan, we have to take 40 ITNs per month. Although ... this will not be enough. And because there is no fuel for this activity, we are obliged to integrate.... Therefore, often we might have a rupture in stock." (FGD ICPs intervention area A)

The district pharmacist was responsible for the stocking of the ITNs for the free distribution at the district level and their disbursement to the CSPS staff when coming to the district to refill their stocks. He was not scared of potential stock ruptures at the district level and saw them as something sufficiently cared for.

R: "At my level, I can not say that there are difficulties, ...stock ruptures were imminent but luckily people thought of that. I can not say that they [stock ruptures] are a problem, ... the additional stocks arrived... there is no problem for the time being at my level." (II district pharmacist)

## Social marketing

### Rural shopkeepers and their decision to sell or not to sell Serena ITN

Of the six rural shopkeepers interviewed, four were aware that the subsidized ITN Serena existed. They knew Serena either from the social marketing (SM) campaign activities of PSI in the villages or from the wholesaler in Nouna town, who had also promoted the ITNs to his rural shopkeeper clients. They had a positive opinion on the SM campaign and its ITN promotion visits. The product was perceived as of good quality. Three of these four shopkeepers agreed to sell the subsidized ITNs. The good quality of the product contributed to their decision to sell the Serena ITNs. Another reason for agreeing to sell the ITNs was the fact that this product improves the health of the people.

R: "I accepted to sell the ITN because it is of good quality and it is something that protects people. So, if it protects us all and gives us health, that is what is good. It is for this reason that we sell the ITNs so that everybody benefits." (ITN selling shopkeeper village Werebere)

R: "I wish that everybody can benefit [from an ITN] to avoid mosquitoes. Because it looks like that the mosquitoes transmit too much malaria to the small children." (ITN selling shopkeeper village Dara)

Two of the six shopkeepers had not heard of the SM campaign and did not sell ITNs. One of them had never seen ITNs being sold in shops like his and, therefore, did not know that it was possible to sell ITNs in his shop. The other one said that ITNs were not demanded in his shop and were not a product easy to sell. The shopkeeper who knew of the SM campaign, but refused to sell the Serena ITNs stated that need is there, but no money to purchase ITNs. Likewise, the wholesaler also stated that demand for ITNs is there, even from other provinces. However, he stated that some of his shopkeeper-clients are reluctant to buy Serena ITNs because they fear that they will not be able to sell Serena ITNs as villagers do not know the product.

*R: "The problem we had, was at the level of our clients. If we say to our clients to buy ITNs, some of them will answer that in their village, people do not know *Serena *and that if they pay now, in their village people will not pay. That was the problem we had." (wholesaler Nouna town)*

The shopkeepers and also the wholesaler did not see the FD as a threat to their sales. One reason for this was that they sell to a wider audience than that concerned by the FD. One shopkeeper even stated that the FD could have a positive, promotional effect on the sales: the pregnant woman receiving a free ITN experiences the positive effects of the ITN and can decide to buy an additional ITN for other children living in the family.

Also the two promoters of PSI were not of the opinion that the free distribution interfered with their social marketing of the ITNs.

R1:" No, this had no influence. I knew that there were 5000 ITNs to be given to pregnant women and children below 5 years of age in the CSPS. When I enter a shop and the shopkeeper is already informed about that [the free distribution] I tell him that these ITNs are for the pregnant women and children below 5 years of age.... It is not about us, our work....we, all the others [the rest of the population], we can have the ITNs for 1500 FCFA." (promoter 1, PSI)

R2: "We did the social marketing in the area, but I never experienced problems because ITNs are distributed for free, no." (promoter 2, PSI)

### Knowledge of and opinions on the price

The shopkeepers who had agreed to sell Serena ITN and the wholesaler were aware of the official price of the ITN for the final customer (1500 FCFA). This was perceived by the shopkeepers as either fair or too expensive for the very poor population. Therefore, they would like to have the price kept or decreased, but not increased. Contrary to the shopkeepers, the director of the SM programme regards the price as too low which might lead to net leakage as it attracts sellers from more distant areas.

R: "... either they go to Mali or they go to Nouna or they go to the next largest town after Nouna., I can imagine that there is somebody there, who buys big quantities to serve the two or three streets passing through this area... tradesmen are very dynamic.... If they see that they can buy an ITN of this quality for 1,500 FCFA, well, if it would be me, I would go there trying to buy to do business.... Maybe it was too cheap." (Director SM)

### Net leakage

The director of the SM programme imagined the possibility that Serena ITNs could be sold to neighbouring countries, e.g. Mali, as demand is existing. He assumed that if people would become aware that in Burkina Faso, ITNs are available on the market and sold, they would either come to Burkina to buy the ITNs or Burkinabé merchants would go to Mali to sell ITNs there.

R: "... because the sold ITNs [in Mali]... it is the same background... the ITN distributed in Mali up to the border of Burkina. They sold it in the prenatal clinics, it was sold to the women for 500 FCFA. But this leaves a whole other population out, other than the pregnant women, the small children who want an ITN. So it would not surprise me that people from the other side of the border have heard of the ITNs ... in Burkina for 1500 FCFA... they will buy them, even the tradesmen from here [Burkina Faso] go there [Mali]." (Director SM)

Although the wholesaler stated not having sold any Serena ITNs to clients from distant areas, as the ITNs of the intervention were reserved for the Kossi province, he highlighted that in fact he also had had demands from distant areas, i.e. other provinces.

R: "We too, we have refused to sell the ITNs out of our province. Because they sold us/our province the ITNs, if somebody comes from outside, we refuse.... the people ask....the people leave other provinces. For example Dedougou. Dedougou had ITNs even before us, they know [the ITNs], even Ouagadougou, Bobo Dioulasso, if there are no more ITNs there, they ask us." (wholesaler Nouna town)

### The marketing relationship between shopkeepers and wholesaler

Limited knowledge on the SM campaign was a problem when it came to refilling the stocks. In spite of having been advised to do so during the ITN promotion visits, none of the shopkeepers interviewed visited the wholesaler in Nouna town to refill their stocks. Either because they had no, or only a vague idea of the wholesaler's role in Nouna town. One shopkeeper feared that other shopkeepers had already refilled their ITN stocks and therefore he would not be able to sell more ITNs. As a consequence, he did not refill his stock. One seller also mentioned that if you do not know the wholesaler beforehand, there would be the danger that he will demand a high price.

R: "...if you see [the ITN] with somebody [a salesman] you do not know, you cannot talk so much. If you say that you want to buy a whole stock, he will still demand from you a high price per piece. And like this, you will not be able to resell it." (not ITN selling shopkeeper village Dara)

One shopkeeper admitted his fear that wealthier shopkeepers could buy large amounts of ITNs to resell them to smaller shopkeepers at a higher price.

R:"... the salesmen who have money are going to buy a lot and when there is nothing left for you, if you want to buy from them, they will charge a higher price." (ITN selling shopkeeper village Dembo)

A relationship between the wholesaler and the shopkeepers on the basis of trust, especially regarding prices, is seen as a prerequisite for a longstanding relationship by all parties. When there is no good relationship, no trust, this is seen as disadvantageous.

R: "... you cannot send goods simply like that, without trust between the person and you. You must find salesmen with whom you have good relationship." (wholesaler Nouna town)

## Discussion

This qualitative study investigates providers' knowledge and perceptions of two alternative ITN delivery interventions in a rural area of Burkina Faso. It contributes to a deeper understanding of which factors may enhance or hinder acceptance of such programmes, which is relevant to secure a sustainable programme implementation. The two intervention programmes implemented were free distribution of ITNs to pregnant women attending ANC services and social marketing of ITNs to the general populations.

Results showed that regarding the FD intervention the related programme and working strategies were known to the people involved. This can already be seen as an achievement of the programme. However, regarding the SM intervention knowledge gaps became apparent: not all the shopkeepers interviewed were aware of their potential role as Serena ITN retailers and of the SM campaign. This could be seen as the main reason why shopkeepers did not participate in retailing the Serena ITNs. A programme on community health insurance schemes in Uganda identified lack of basic information about the scheme as one factor leading to low participation in the scheme [35]. In the current study in Burkina Faso it shows that the social marketing campaign including the ITN promotion visits of PSI was not sufficient. However, as PSI had not worked in the NHD before it may take some more time to establish a functioning market and retail scheme. PSI financed their running costs from the revenues of the social marketing sales, if there would have been additional funds available a prolonged and more intense social marketing campaign would have been possible.

The CSPS staff appreciated the FD intervention. They saw the intervention as an aid supporting the poor population, a means of improving the health of the community, and of increasing overall ANC attendance. Interestingly, data on ANC visits in the intervention area showed increasing numbers of ANC attendance over the years. However, no difference was observed in the two intervention arms. This showed that the increase is part of an overall trend and can in reality not be linked to the FD intervention, in spite of the fact that the health staff perceived it that way [unpublished findings]. The rural shopkeepers appreciated the SM intervention, if it was known to them. Interestingly, the FD intervention in the CSPS was not seen by the shopkeepers as a threat to their sales. Müller *et al *[34] showed that there was an increase in ITN ownership in both intervention areas, providing evidence that FD does not necessarily interfere with sales. This would mean that people purchase ITNs via local shops even though ITNs are distributed for free to pregnant women via ANC services.

Compliance with the implementation of the intervention and motivation among CSPS staff was high. This might result from the positive view of the beneficial impact of the intervention on the community. Health staff felt that their attractiveness and 'power' with the community increased through the FD intervention. The fact that a project continuation (intervention area A) or project expansion (intervention area B) was desired by the CSPS staff despite the additional workload further stresses the success of the programme. The argument that ITN distribution through ANC did not constitute an unbearable burden for the local health staff is further backed by the fact that existing resources are currently utilized well below their potential [36,37].

The CSPS staff perceived the distribution procedure of the ITNs as transparent. They did not cite any problems regarding misuse or double giving of ITNs. On the SM side there were no hints on price abuse: the wholesaler and the shopkeepers who sold Serena had a clear knowledge of its price and appeared to have adhered to it. A qualitative study involving the communities in the intervention area supports these findings [27]. There were neither reports on incorrect distributions of ITNs happening nor price abuse. Also in a social marketing programme in Tanzania retailers generally adhered to the product price [38]. However, there are reports on abuse of e.g. voucher schemes in other countries [39]. The responsible of PSI and the wholesaler in Nouna town said that there exists demand for ITNs in other regions and/or countries. The wholesaler denied selling ITNs to shopkeepers from other regions. However, as by market law products go where there is demand for them, ITN leakage to distant regions/countries is possible. There is evidence from other SSA countries as well, that unsaturated markets surrounding the intervention province trigger net leakage of highly subsidized ITNs [39,40]. This rather talks against using market mechanisms only to increase ITN coverage and supports the approach of complementing market sales with free distribution programmes.

The problem related to the randomized design of the study (i.e. pregnant women from intervention area B switching to a CSPS of intervention area A) should now be solved as FD has become a national policy in Burkina Faso. An unresolved problem is that, according to the CSPS staff, the distribution of the ITNs at the 1^st ^ANC visit of the women makes women come earlier - but then the women do not come back for the following ANC visits once they have got the ITN. In Nouna region, currently only 18.5% of women complete all four WHO recommended ANC visits [41]. However, for protection of the pregnant woman the ITN must be given at the first ANC visit and cannot be used as an incentive later to increase the amount of ANC visits completed. As this was a suggestion made by the CSPS staff the necessity of protection of the pregnant woman as early as possible should be reemphasized again during training and supervision.

The biggest problem mentioned by the CSPS staff was the stock ruptures at the level of the CSPS. As an intervention that is well accepted cannot afford logistic breakdowns, the nature of distribution within the system needs to be improved. Other studies report that frequent unavailability of necessary equipment can have negative impact on motivation and performance of health workers [42-44]. To keep the motivation of the health staff towards the FD at high levels, an unavailability of ITNs must be avoided. The decision that health staff should refill their stock at the central deposit of the District located in Nouna town was made in order to keep costs low, i.e. avoid additional travels to Nouna just for the purpose of refilling the ITN stock. However, to avoid stock ruptures additional travels seem to be necessary. Alternatives to circumvent the stock rupture problem could be budgeting for additional travels of the health staff to Nouna town or delivering the ITNs with a car to the CSPS, e.g. once a month. The car would also have more transport capacity than the motorbikes. However, the problem with bad road conditions, especially in the rainy season, stays.

In marketing there is always a risk and some insecurity. Additionally sales require that there is not only knowledge of and a need for a product, but also demand, i.e. a capacity to pay to meet that need. Shopkeepers stated that in the rural villages demand is either not there, or demand is there but there is no capacity to buy the ITNs as the population is too poor. This argument is strengthened by a qualitative study involving the communities of the intervention areas, which showed that prices are perceived as too high [27]. Shopkeepers feared the risk of unsold ITNs due to the low capacity to pay for the product in the community. This and the fact that shopkeepers were not aware of the details of the SM campaign or where to refill their stocks could be named as the major problems emerging. Trust between the shopkeepers and the wholesaler seems to be a major characteristic for the marketing relationship between the two to be sustainable.

The study has some limitations, e.g. in the process of translation of the interview guide from French into Dioula and vice versa (for the interviews with the rural shopkeepers and the wholesaler in Nouna town), and the afterward transcription of interviews, the possibility exists that important information got lost or emphases in interviewees' responses were accidentally changed. This might have had an influence on the transcription and translation of the interviews hence leading to slightly different results and/or interpretation. However, the interviewers' training process should have taken sufficiently care of this. Furthermore the first author is not a French native speaker and in the interviews conducted by her, misunderstanding could have been occurred. To ensure consistency in the translation, the transcription and translation of randomly selected passages from the interviews was checked by the third author. Finally, the analysis was triangulated at various stages by the first, second, and third author in order to avoid misunderstandings and to ensure a good coding scheme.

The number of shopkeepers interviewed can be seen as too low considering the scope of the intervention. For convenience the interviews with the rural shopkeepers were conducted in villages where FGDs with the community had taken place in the framework of the overall community-based cluster-randomized controlled trial [see Ref. [27] for details]. Unfortunately in one village there existed only one shop and in another village there was nobody selling the Serena ITN, which unexpectedly limited the number of interviews with shopkeepers.

## Conclusion

The combination of ITN free distribution and social marketing was in general well accepted by the different providers. However, low purchasing power of clients and the resulting financial insecurities of shopkeepers remain a challenge to ITN social marketing in rural SSA.

## Competing interests

The authors declare that they have no competing interests.

## Authors' contributions

CB: contributed to the conception and design of the study, developed the questionnaire, collected the data, analysed the data and wrote the paper: MDA: contributed to the conception and design of the study, developed the questionnaire, contributed to analysis and interpretation of the data and to writing the paper; JT: contributed to developing the questionnaire, collected the data and to writing the paper; MY: contributed to designing the study and to writing the paper; AJ: contributed to designing the study, interpretation of the data and to writing the data; OM: initiated the conception and design of the study and contributed to interpretation of the data and to writing the paper; All authors read and approved the final manuscript.
